# Predictors of mothers’ preventive behaviors for children’s dental trauma: a cross-sectional study using the health belief model

**DOI:** 10.1038/s41405-025-00346-4

**Published:** 2025-06-12

**Authors:** Esmaeil Fakharian, Mojtaba Sehat, Azam Jahangirimehr, Hossein Akbari, KHadijeh Kalanfarmanfarma, Soudabeh Yarmohammadi

**Affiliations:** 1 Trauma Research Center, Department of Neurosurgery, Kashan University of Medical Sciences, Kashan, Iran; 2 MD, PhD in Epidemiology, Trauma Research Center, Department of Community Medicine, Faculty of Medicine, Kashan University of Medical Sciences, Kashan, Iran; 3 Shoushtar Faculty of Medical Sciences, Shoushtar, Iran; 4 Associate Professor of Biostatistics, Social Determinants of Health (SDH) Research Center, Kashan University of Medical Sciences, Kashan, Iran; 5 Trauma Research Center, Kashan University of Medical Sciences, Kashan, Iran

**Keywords:** Dental trauma, Dentistry

## Abstract

**Objective:**

Dental traumas are one of the most common reasons for children to visit the dentist. This study aimed to investigate the predictors of mothers’ behavior based on the health belief model (HBM) for the prevention of dental trauma in 7–12-year-old children.

**Methods:**

The current cross-sectional study was conducted on 700 mothers in Kashan city in 19 September 2023 to 2 March 2024 included in the study by multi-stage random cluster sampling method. The data collection tool was a valid and reliable researcher-made questionnaire consisting of demographic information, Knowledge, constructs of HBM, and preventive behavior for dental trauma. Data were analyzed using SPSS 21 and descriptive statistics (standard deviation, mean, median and range), inferential tests (Pearson correlation coefficient, regression) and path analysis to test the direct and indirect effect of model constructs on the dependent variable by AMOS software.

**Results:**

Knowledge (*r* = 0.365, *P* < 0.001), perceived benefits (*r* = 0.166, *P* < 0.001), and self-efficacy (*r* = 0.425, *P* < 0.001) had a positive correlation and perceived barriers (*r* = −0.313, *P* < 0.001) had a negative correlation and a significant relationship with mothers’ preventive behaviors. Knowledge, perceived barriers and self-efficacy explained and predicted a total of 33% of preventive behavior changes (R-Square=0.329). In the path analysis, self-efficacy (*β* = 0.327, *P* < 0.001), knowledge (*β* = 0.251, *P* < 0.001) and perceived barriers (*β* = −0.242, *P* < 0.001) had the most direct effect, and perceived severity (*β* = −0.017), perceived susceptibility (*β* = −0.004), and perceived benefits (*β* = 0.092) had an indirect effect on mothers’ preventive behaviors (*P* < 0.05).

**Conclusion:**

Knowledge, self-efficacy, and perceived barriers were key predictors of mothers’ preventive behaviors. Interventions should target these factors to improve dental trauma prevention in children. The HBM effectively identified these predictors.

## Introduction

Traumatic injuries not only represent a global health risk but also rank among significant social issues [1]. Dental trauma is one of the important categories, which accounts for the majority of health problems in children and adolescents [2]. Epidemiological studies indicate that the global annual incidence of dental trauma is approximately from 1 to 44 new cases per 1000 persons. prevalence of dental injuries occurs at varying rates, ranging from 6% to 59%. About one-third of children with primary teeth and one-fifth of teenagers and adults with permanent teeth have experienced traumatic dental injuries [3].

Traumatic dental injuries in primary teeth are a significant public health concern, particularly among preschool and school-aged children. Epidemiological studies highlight their high prevalence, with approximately 15% of children under 6 years and 12% of those over 6 affected, primarily due to falls occurring at home [4]. In school-aged children, the prevalence rises to around 25% [5], with falls and play-related accidents being the most common causes; tooth number 21 is frequently involved, and schools are often the location of injury [6]. Preschool children are especially vulnerable due to their active growth stage and increased fall risk [7]. The consequences of TDIs extend beyond immediate physical damage. Cortes et al. (2017) reported that 15% of children aged 2–5 years with dental trauma developed aesthetic complications such as tooth discoloration [8]. These injuries are also associated with substantial treatment costs, functional impairments, and potential long-term sequelae [9]. Furthermore, pain, discomfort, and the visible nature of dental injuries can negatively impact a child’s quality of life [10], underscoring the need for early intervention and prevention strategies.

Previous studies have examined parents’ knowledge and management of dental trauma across various countries and demographics [11, 12]. Momeni et al. [13] found that few Iranian mothers were aware of traumatic dental injuries and did not recognize dental trauma as a significant health concern [13]. In the study conducted by Rouhani et al. [14], reported that 178 children experienced trauma to their permanent teeth, with maxillary central incisors being the most frequently affected (84%). The main causes of dental injuries identified were falls (42.9%) and fighting (34%), with most incidents occurring at home (46.8%) and at school (34%) [14].

Given that most dental injuries in children occur at home, parents play a crucial role in managing these incidents [15]. Their understanding of how to control and treat trauma is vital for the long-term success of dental interventions and significantly impacts overall oral and dental health. Consequently, preventing these injuries largely relies on parents’ knowledge, particularly that of mothers [13]. Developing programs to prevent dental injuries presents a challenge for authorities, dental associations, and health professionals [16]. Creating evidence-based preventive strategies requires a thorough understanding of the multifaceted causes of dental injuries in children, encompassing social, behavioral, and biological factors [17]. Health education is one of the important activities that are typically conducted to promote health. It consists of information that enables people to make informed decisions about their health. The value of educational programs depends on their effectiveness, which is primarily related to the correct use of theories and models in health education [18].

## Health belief model (HBM)

The HBM is a widely used psychological theory that examines how individual beliefs influence health-related behaviors. Originally developed for disease prevention, the HBM provides a structured approach to understanding decision-making processes in healthcare. While this model has proven valuable across various fields including chronic disease management, health education, and community intervention assessments researchers emphasize the need for continuous updates to ensure its adaptability to evolving public health challenges [19]. Researchers often employ this model to investigate how people make personal health decisions, ranging from participation in medical screenings to daily oral care routines and management of harmful behaviors [20–22].

Numerous studies have emphasized that the HBM, with its targeted structure and specialized constructs, offers unique theoretical and practical advantages for preventive research, particularly in the field of oral health. This model’s simultaneous consideration of perceived severity and susceptibility and self-efficacy demonstrates superior capability in predicting preventive behaviors compared to more general models. For instance, a systematic review has shown that the application of HBM effectively improves oral health behaviors, highlighting the key roles of constructs such as self-efficacy and perceived threat in behavior change [23].

The HBM comprises six key dimensions that influence health behaviors: perceived susceptibility: An individual’s assessment of their vulnerability to a health threat. perceived severity: The perceived seriousness of a health condition and its potential consequences, incorporating both medical and social impacts. perceived benefits: The perceived effectiveness of preventive measures. perceived barriers: Practical or psychological obstacles preventing recommended health actions. self-efficacy: One’s belief in their ability to successfully perform health-protective behaviors, now recognized as a distinct element of the model. cues to action: Internal or external prompts (e.g., symptoms or health messages) that activate health-protective decision-making [19]. To encourage preventive behaviors among mothers regarding dental trauma in children, they must first recognize their susceptibility to such injuries and comprehend the severity of the consequences, which can include negative physical, psychological, social, and economic impacts. Furthermore, mothers should evaluate the perceived benefits of preventive measures against any perceived barriers, whether they are physical, financial, or mental. Self-efficacy plays a crucial role in this process, as it reflects a mother’s confidence in her ability to engage in preventive behaviors effectively. A strong sense of self-efficacy can motivate mothers to overcome barriers and adopt preventive strategies. Ultimately, this comprehensive understanding can facilitate the adoption of preventive behaviors. However, there is a scarcity of research exploring mothers’ preventive actions for children’s dental trauma through the lens of theories or behavioral models. This study serves as a pioneering effort to identify predictors of mothers’ preventive behaviors based on the Health Belief Model for preventing dental trauma in children. This study highlights that understanding these predictive factors and sharing this knowledge with healthcare policymakers could be a crucial step toward reducing dental trauma injuries.

## Methods

### Research plan

The current cross-sectional study was conducted from September 19, 2023, to March 2, 2024, in Kashan, Isfahan province. As the largest city in Isfahan with a population of 364,482, Kashan served as the study setting. The research population consisted of mothers of children aged 7 to 12 years, who were selected from local schools. The inclusion criteria for the study were as follows: 1. Mothers with children aged 7–12; 2. Mothers residing in the city of Kashan; 3. Mothers reading and writing proficiency for questionnaire completion; 4. Mothers fluent in Persian to ensure proper understanding of study materials; and finally, mothers who were willing to participate in the study. The exclusion criteria were as follows: 1. Mothers of children with minor disabilities or specific medical conditions that could influence the risk of dental trauma; 2. Mothers who did not live in the study area or were temporary residents; and 3. Mothers whose children had previously suffered severe dental trauma that might have impacted their preventive behaviors.

In this study, all methods were carried out in accordance with the Declaration of Helsinki and approved by the ethics committee of Kashan University of Medical Sciences, with the ethics code of IR.KAUMS.NUHEPM.REC.1402.032. All these methods followed relevant guidelines, literature review, and regulations approved by the Kashan University of Medical Sciences, Kashan, Iran. Written informed consent was obtained from all participants prior to taking part in this study.r

### Sample size and sampling technique

This study was conducted with the aim of predicting mothers’ behavior to prevent dental trauma in children. The sample size was estimated based on the following formula.

$${{{\boldsymbol{N}}}}=\frac{{{{{\boldsymbol{Z}}}}}^{{{{\bf{2}}}}}{}_{{{{\bf{1}}}}-\propto /{{{\bf{2}}}}}* {{{\boldsymbol{SD}}}}{{{\bf{2}}}}}{{{{{\boldsymbol{d}}}}}^{{{{\bf{2}}}}}}$$

In related studies, for example, Momeni et al. [24] reported that the score of mothers’ preventive performance of dental trauma was 3.38 ± 1.2. Therefore, if margin of error (*d*) = 0.1, SD = 1.2, and$\,{{{{\rm{Z}}}}}^{2}{}_{1-\propto /2}=1.96$, according to the formula above, the sample size was obtained 554 people. Taking into account a drop-out rate of 20% and above, the sample size was considered 700 people, who were selected based on the multi-stage random cluster sampling method. To gather samples, we focused on mothers of children aged 7 to 12. Initially, we approached the Kashan General Education Department and segmented the Kashan into five geographical regions: north, south, east, west, and center. We treated each of the five regions as a separate cluster, resulting in a total of five clusters. Subsequently, we obtained a list of both girls’ and boys’ schools in each cluster from the Education Department. From each of these clusters, we randomly selected two schools—one for girls and one for boys—culminating in a total of 10 schools. It is important to note that 70 mothers were randomly selected from each school, resulting in a total of 700 completed questionnaires.

### Data collection process

Data were collected in two shifts, morning and afternoon, from 10 schools using a researcher-developed questionnaire by the corresponding author. The corresponding author visited the schools and coordinated with the administrators to obtain the schedule of parent-teacher meetings. On the day of the meetings, after the session concluded, mothers present were asked to stay for 15 to 20 min to complete the questionnaires. Following the introduction of the study and its objectives, the researcher randomly distributed questionnaires among the mothers of children aged 7–12 years. The researcher’s presence at the parent-teacher meetings of each school continued until the sample size was reached. To minimize bias, the researcher read the questions aloud.

### Instrumentation

The researcher-developed questionnaire, grounded in the HBM, comprised three sections. The first section gathered demographic information through 13 questions, which included the child’s gender and age, the mother’s age, the child’s educational level, the mother’s and father’s education and job, the number of children in the family, economic status, housing situation, marital status, and inquiries such as, “Have you previously received information about the prevention of dental trauma?”

The second section comprised nine knowledge-related questions derived from a literature review. The knowledge-based questions (e.g., definition of dental trauma, complications, prevention) were scored based on accuracy. For each item, a “Yes” response was awarded 3 points only if it matched the correct answer derived from the standard dental trauma literature. Incorrect “No” responses received 2 points, while “I do not know” (reflecting lack of knowledge) was assigned 1 point. This scoring system ensured higher scores reflected better knowledge, with total scores ranging from 9 to 27. The third section of the questionnaire addressed the constructs of the HBM. In this study, questions were formulated to assess perceived susceptibility, perceived severity, perceived barriers, perceived benefits, self-efficacy, and behavior.

The perceived susceptibility construct comprised four questions, one of which was, “ likely my child is at risk of breaking a tooth while playing with friends.” Responses were measured using a 5-point Likert scale (strongly agree, agree, have no opinion, disagree, and strongly disagree), with the highest score assigned to “strongly agree” (score of 5) and the lowest to “strongly disagree” (score of 1). The total score ranged from 4 to 20. The perceived severity construct consisted of five questions, one of which was, “If my child’s tooth breaks, she will have difficulty eating properly.” Responses were assessed using a 5-point Likert scale (strongly agree, agree, have no opinion, disagree, and strongly disagree), with “strongly agree” receiving the highest score (5) and “strongly disagree” the lowest (1). The total score could range from 5 to 25.

The perceived benefits construct comprised five questions, one of which was, “Wearing a mouth guard while children play can help prevent tooth fractures.” In contrast, the perceived barriers construct also included five questions, such as, “Using devices to prevent tooth breakage in children may be expensive for families.” The self-efficacy construct included five questions, one of which was, “I can encourage my children to wear mouthguards while exercising.” The Likert scale for the perceived benefits and self-efficacy constructs was administered separately, using a 5-point scale (strongly agree, agree, have no opinion, disagree, and strongly disagree), where “strongly agree” received the highest score of 5 points and “strongly disagree” received the lowest score of 1 point. For the construct of perceived barriers, the scores were applied in reverse, that is, the highest score was given to strongly disagree (score 5) and the lowest score was given to strongly agree (score 1). The score range of all three constructs was between 5 and 25.

Five questions were created to assess mothers’ preventive behaviors regarding children’s dental trauma. One example question was, “Have you ever advised your children not to push each other while playing?”. Responses were measured using a 4-point Likert scale (never, sometimes, often, and always), with the highest score of 4 points assigned to “always” and the lowest score of 1 point given to “never.” The total score ranged from 4 to 16.

### Validity and reliability of the instrumentation

To create the questionnaire, initial library research was conducted on the topic [25–27]. Face-to-face interviews were held with 10 mothers of children aged 7–12 (who did not participate in subsequent phases of the study) to assess the qualitative face validity. They were asked about the difficulty level, appropriateness, and clarity of the questionnaire items. To evaluate the quantitative content validity of the tool, 10 experts in dentistry, health education, and nursing were consulted regarding the necessity, relevance, simplicity, and clarity of each question. Based on Lawshe’s criteria [28], for a panel of 10 experts, items with a Content Validity Ratio (CVR) exceeding 0.62 are deemed statistically significant. In the present study, the calculated CVR of 0.79 meets this threshold, confirming the essentiality of the included items. For the Content Validity Index (CVI), we applied the method proposed by Waltz and Bausell [29], where scores below 0.70 are considered unacceptable. Our obtained CVI of 0.72 surpasses this cutoff, demonstrating adequate content validity for the overall questionnaire.

Cronbach’s alpha coefficients were computed for knowledge (0.79) and various constructs of the HBM, including perceived susceptibility (0.78), perceived severity (0.83), perceived barriers (0.71), perceived benefits (0.85), and self-efficacy (0.82). The reliability of the questionnaire was assessed using a test-retest method. In this process, 30 eligible mothers completed the questionnaire twice, with a two-week interval between administrations, and the scores from both stages were compared. The reliability coefficient was found to be 0.8.

### Data analysis

Quantitative variables were summarized using descriptive statistics, including standard deviation, mean, median, and range, while qualitative variables were reported as frequencies and percentages. Pearson’s correlation coefficient was utilized to create the correlation matrix for the model constructs, and multiple linear regression analysis was employed to examine the relationships among the model constructs, knowledge, and behavior. Prior to regression analysis, key assumptions were checked: (1) Linearity was assessed using scatterplots of residuals and partial regression plots; (2) No missing data were observed in this study, and sampling proceeded until the predetermined sample size was attained; and (3) Multicollinearity was evaluated using variance inflation factors (VIFs), with all values below 3 indicating no concerns. In constructing the regression model, the order of variable entry was determined through a combination of empirical and statistical considerations. First, we examined the bivariate correlations between independent variables and the outcome, prioritizing variables with stronger associations (*p* < 0.2) for initial inclusion. A stepwise regression approach was then applied to select the most significant predictors, ensuring an optimal balance between empirical data and the Health Belief Model framework. Demographic variables were examined as potential covariates in preliminary analyses. However, none showed statistically significant associations with mothers’ preventive behaviors in our sample (*p* > 0.05). Consequently, these variables were not included as covariates in the final regression model to maintain parsimony.

Path analysis was conducted to assess both the direct and indirect effects of the model constructs on the dependent variable. For data analysis, AMOS and SPSS21 software were used, with a significance level set at *p* < 0.05.

## Results

### Sample characteristics

The study achieved a 0.0% non-response rate, as all participants completed the questionnaire without any missing data. The results indicated that majority of the mothers were aged between 30 and 40 years (68.3%), with 51.4% of the children being girls and 48.6% boys. Additionally, 47% of the mothers held associate or bachelor’s degrees, 69% were homemakers, 42% reported an average economic status, and 72% had knowledge about preventing dental trauma in children (Table 1).

**Table 1:** Demographic variables information (*N* = 700).

Variable	Categories	Frequency(*n*)	Percent (%)
Gender of the child	Female	360	51.4
	Male	340	48.6
Age of the child (years)	7–10 (y)	319	45.6
	10–12 (y)	381	54.4
Age of the mother (years)	20–30 (y)	36	5.1
	30–40 (y)	478	68.3
	40–50 (y)	186	26.6
Educational level of the child	First-grade	76	10.9
	Second- grade	110	15.7
	Third -grade	91	13.0
	Fourth-grade	106	15.1
	Fifth-grade	210	30.0
	Sixth- grade	107	15.3
Educational level of the mother	Below diploma/diploma	319	45.6
	associate/bachelor’s degree	330	47.1
	Master’s Degree	43	6.1
	Ph.D./Physician	8	1.1
Number of children in the family	1–2 child	518	74.0
	3–4 child	182	26.0
Mother job	Employee	153	21.9
	freelance job	63	9.0
	housekeeper	484	69.1
Marital status	Married	674	96.3
	Divorced/Widow	26	3.7
Educational level of the father	Below diploma/diploma	353	50.4
	associate/bachelor’s degree	263	37.6
	Master’s Degree	74	10.6
	Ph.D./Physician	10	1.4
Father job	Employee	265	37.9
	freelance job	421	60.1
	Not working	12	1.7
	Separated/Deceased	2	0.3
Economic status	Upper	164	23.4
	Middle	297	42.4
	Lower	239	34.1
The state of the house	Owners	522	74.6
	Renter	178	25.4
Information about dental trauma prevention	Yes	504	72.0
	No	196	28.0

Table 2 explains some of the central indicators and dispersion of knowledge, HBM constructs, and mothers’ preventive behavior variable. Mean and standard deviation of knowledge included (22.028 ± 4.151), perceived susceptibility (11.240 ± 3.398), perceived severity (18.765 ± 3.289), perceived benefits (19.818 ± 2.909), perceived barriers (16.655 ± 3.118), self-efficacy (20.610 ± 2.925), and preventive behavior (14.755 ± 2.407). The findings of this study revealed a significant correlation between knowledge and constructs (perceived benefits, perceived barriers, and self-efficacy), except for the constructs of perceived severity and susceptibility, with mothers’ preventive behaviors (Table 3).

**Table 2:** The mean scores of variables (*N* = 700).

Variable	Mean ± SD	Median	Score range
Knowledge	22.028 ± 4.151	23.000	0–27
Perceived susceptibility	11.240 ± 3.398	11.000	4–20
Perceived severity	18.765 ± 3.289	19.000	5–25
Perceived benefits	19.818 ± 2.909	20.000	5–25
Perceived barriers	16.655 ± 3.118	17.000	5–25
Self-efficacy	20.610 ± 2.925	20.000	5–25
Preventive behaviors of DT	14.755 ± 2.407	15.000	5–20

*DT* dental trauma.

**Table 3:** The correlation coefficient between the HBM constructs and DT preventive behaviors, Pearson’s method.

Variables	1	2	3	4	5	6	7
1 Knowledge r (*p*)	1	–	–	–	–	–	–
2 Perceived susceptibility *r* (*p*)	0.047 (0.213)	1	–	–	–	–	–
3 Perceived severity *r* (*p*)	0.115 (0.002)	0.282(<0.001)	1	–	–	–	–
4 Perceived benefits *r* (*p*)	0.224(<0.001)	0.207(<0.001)	0.415(<0.001)	1	–	–	–
5 Perceived barriers *r* (*p*)	−0.114(0.003)	0.171(<0.001)	0.204(<0.001)	0.058(0.124)	1	–	–
6 Self-efficacy *r* (*p*)	0.264(<0.001)	0.048(0.209)	0.136(<0.001)	0.316(<0.001)	−0.128(<0.001)	1	–
7 Preventive behaviors of DT	0.365(<0.001)	0.010 (0.789)	0.034 (0.365)	0.166(<0.001)	−0.313(<0.001)	0.425(<0.001)	1

*DT* dental trauma.

The simultaneous effect of knowledge and HBM constructs on the dependent variable of mothers’ preventive behaviors was investigated by Multiple Linear Regression analysis model (*P* > 0.200). The independent variables were entered into the regression equation step by step in the order of their importance in explaining the dependent variable. The final model had a good fit (*F* = 102.151, *P* < 0.001). Collinearity results showed that there is no collinearity between independent variables (Table 4). The three constructs of knowledge, perceived barriers and self-efficacy were able to explain and predict 33% of preventive behavior changes (R-Square = 0.329), and self-efficacy showed to be the strongest predictor of the mothers’ preventive behavior (*B* = 0.328). The normality of the residuals was tested using the Shapiro-Wilk test, which showed that the residuals were normally distributed (*p* value = 0.105) (Table 4).

**Table 4:** Examining the relationship between HBM constructs and DT preventive behaviors, a Multiple Linear Regression analysis.

Variable	Unstandardized coefficients		Beta standardized	*t*	Sig	95.0% confidence interval		Collinearity Statistics		R-square
	*B*	Std. error				Lower bound	Upper bound	Tolerance	VIF	
(Constant)	9.110	0.787		11.576	0.000	7.565	10.655			0.329
Self-efficacy	0.270	0.027	0.328	9.952	0.000	0.216	0.323	0.920	1.086	
Knowledge	0.145	0.019	0.251	7.630	0.000	0.108	0.183	0.924	1.083	
Perceived barriers	−0.187	0.025	−0.242	−7.572	0.000	−0.235	−0.138	0.977	1.024	

### Path analysis

**Fig. 1:**
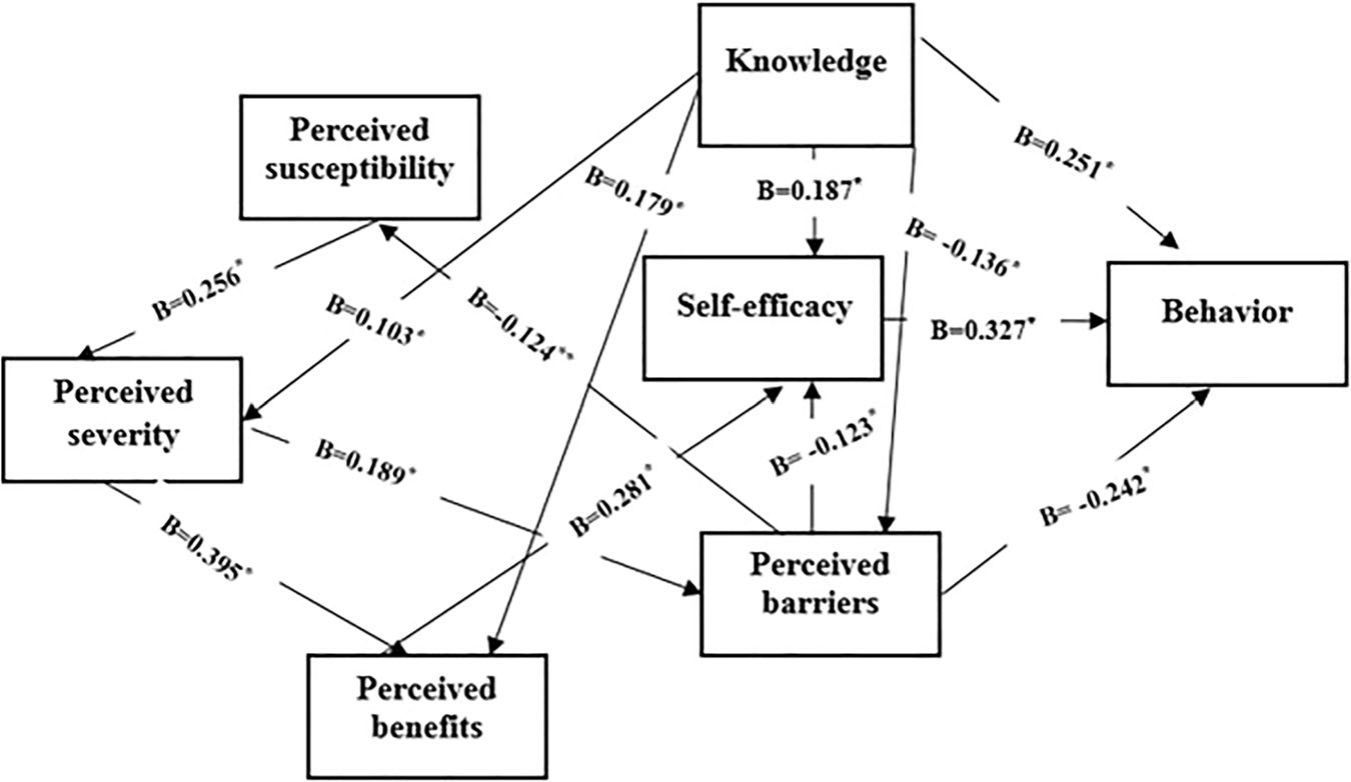
The relationship between knowledge and HBM constructs and DT preventive behavior of mothers. *P < 0.001, **P < 0.05.

The results of this analysis showed that knowledge (*β* = 0.251, *P* < 0.001), perceived barriers (*β* = −0.242, *P* < 0.001) and self-efficacy (*β* = 0.327, *P* < 0.001) had the greatest direct impact on mothers’ preventive behaviors of their children’s dental trauma, while perceived severity, perceived susceptibility, and perceived benefits did not have a direct effect on mothers’ preventive behaviors. However, these three variables had an indirect effect on mothers’ preventive behaviors. Furthermore, the direct, indirect, and total effects were investigated and the results showed that the knowledge construct had the strongest total effect (*B* = 0.365, * P* < 0.001), self-efficacy (*B* = 0.327, *P* < 0.001) and perceived barriers (*B* = −0.283, *P* < 0.001) (Fig. 1, Table 5). In general, knowledge and the HBM constructs explain 31% of the total variance in mothers’ preventive behaviors (*R*^2^ = 0.306) (Tables 6, 7). The model fit indices also confirmed the appropriateness of the model (Table 7).

**Table 5:** Exploring direct and indirect effects of HBM constructs on DT preventive behaviors, Path analysis.

Variable	Direct effects	Indirect effects	Total effects
Knowledge	0.251	0.114	0.365
Perceived severity	0.000	−0.017	−0.017
Perceived barriers	−0.242	−0.041	−0.283
Perceived susceptibility	0.000	−0.004	−0.004
Perceived benefits	0.000	0.092	0.092
Self-efficacy	0.327	0.000	0.327

*DT* dental trauma.

**Table 6:** Exploring path coefficients and explained variance of HBM constructs and DT preventive behaviors.

Path			*β*	S. E	C.R	*P* value
Perceived barriers	<---	Knowledge	−0.136	0.028	−3.677	<0.001
Perceived severity	<---	Knowledge	0.103	0.029	2.842	0.004
Perceived benefits	<---	Perceived severity	0.395	0.030	11.626	<0.001
Perceived benefits	<---	Knowledge	0.179	0.024	5.257	<0.001
Self-efficacy	<---	Perceived benefits	0.281	0.036	7.846	<0.001
Self-efficacy	<---	Perceived barriers	-0.123	0.033	−3.508	<0.001
Self-efficacy	<---	Knowledge	0.187	0.025	5.202	<0.001
Behaviors	<---	Perceived barriers	-0.242	0.025	−7.589	<0.001
Behaviors	<---	Self-efficacy	0.327	0.027	9.973	<0.001
Behaviors	<---	Knowledge	0.251	0.019	7.647	<0.001
Perceived barriers	<---	Perceived severity	0.189	0.036	4.955	<0.001
Perceived severity	<---	Perceived susceptibility	0.256	0.036	6.942	<0.001
Perceived susceptibility	<---	Perceived barriers	0.124	0.043	3.164	0.002

*DT* dental trauma, *C.R.* critical ratio.

**Table 7:** Goodness of fit indices for the predictive model of DT preventive behaviors.

Fitness indicators	Χ2	DF	Χ2/DF	TLI	NFI	RMR	CFI	IFI	RMSEA	R^2^
Domain	11.720	8.000	1.460	0.985	0.982	0.086	0.994	0.994	0.026	0.306

*Χ2* Chi-square, *TLI* Tucker-Lewis index, *NFI* normed fit index, *RMR* root mean square residual, *CFI* comparative fit index, *IFI* incremental fit index, *RMSEA* root mean square error of approximation.

## Discussion

Due to the lack of theory-based evidence about health beliefs related to mothers’ decision-making and preventive behaviors regarding children’s dental trauma, there is limited information about how beliefs affect mothers’ preventive behaviors regarding children’s dental trauma. Without these data, it is not possible to design interventions based on effective evidence. This study was conducted with the aim of the predictors of mothers’ behaviors the preventive for dental trauma of 7–12-year-old children based on HBM. The results of this study showed that there was a correlation between knowledge and the HBM constructs, except for the perceived severity and susceptibility construct, with mothers’ preventive behaviors for children’s dental trauma. These results show that knowledge and health beliefs can play an important role in forming and changing mothers’ preventive behaviors regarding the prevention of dental trauma in their children.

A study by Silva et al. [30], reported that increasing mothers’ knowledge increases the behavior of preventing accidents in childhood [30]. Similarly, Amini et al. [31] highlighted the significant effect of mothers’ understanding on their ability to prevent accidents involving their children [31]. Knowledge about oral and dental hygiene plays a crucial role in improving children’s oral health, and better education and practices among parents lead to better oral health outcomes for their children [32]. Gudipaneni et al. [33] further noted that educated mothers are more likely to take their children to dental appointments [33]. Effective strategies to increase mothers’ knowledge and promote preventive behaviors include oral and dental health education, health counseling, and the cultivation of an appropriate cultural context. However, simply increasing awareness is insufficient to change behavior. In addition to enhancing knowledge, interventions must also address individuals’ beliefs to facilitate behavioral change.

In this study, there was a correlation between the perceived benefits mothers’ preventive methods to reduce their children’s dental trauma, and preventive behavior. In the study by Heidarikia et al. [34], noted that even before the intervention, had mothers’ a high awareness of the benefits of preventive behaviors, indicating they understood the importance of safe practices [34]. Conversely, Jiang et al. [35], found no significant association between perceived benefits and mothers’ intentions to engage in preventive behaviors for their children’s myopia [35]. In our dental trauma context, mothers who recognized greater benefits (whether physical, social, economic, or psychological) were more likely to adopt preventive behaviors, suggesting that benefit perception may be particularly influential for oral injury prevention.

In this study, perceived barriers construct and self-efficacy had the strongest effect on mothers’ preventive behaviors for children’s dental trauma. One of the perceived barriers was physical, psychological, and financial costs of mothers for preventive behaviors against dental trauma in children. Perceived barriers are one of the important factors of preventive behaviors, so that with the reduction of barriers, there is an increase in preventive behaviors. It is required to provide appropriate training to improve mothers’ intervention techniques and strategies for children and support parents to spend more time helping children to adopt dental trauma preventive behaviors. Ashoori et al. [27] reported that perceived barriers and self-efficacy showed a significant correlation with behaviors and were most related to oral and dental health [27]. Moreover, in the study by Goodarzi et al. [36], the important predictors of oral and dental health were reported self-efficacy, perceived barriers, perceived benefits, and cues to action [36]. In the study by Kamal et al. [37], it was also reported that financial costs were the biggest health barriers [37].

In this study, the reduction of perceived barriers increased mothers’ self-efficacy for preventive behaviors against children’s dental trauma. Self-efficacy plays a positive role in performing health promotion behaviors [38]. Moridi et al. [39], found that higher self-efficacy increased children’s preventive behaviors in accidents [39]. Similarly, Wilson et al. [38] reported that mothers with higher education perceived their children as more vulnerable to dental caries, saw more benefits and fewer obstacles in recommended oral hygiene practices, and had greater confidence in their ability to perform these behaviors [38]. To improve self-efficacy, strategies such as verbal persuasion, role modeling, and practice with failure can be used as a natural part of learning process [40]. Therefore, developing and implementing interventions to enhance mothers’ self-efficacy in managing their children’s oral health is essential.

Mothers’ knowledge about dental trauma, causes of trauma and prevention methods did not increase their perceived ability to perform preventive behaviors. In this study, no correlation was found between the perceived susceptibility and preventive behaviors of mothers towards their children’s dental trauma. Mothers believed that if they do not take preventive actions for their children’s dental injuries, they will not suffer harmful injuries. Jaras et al. [41] reported that perceived susceptibility was the most effective factor affecting mothers’ behaviors regarding the prevention of accidents and incidents in children [41]. Similarly, Moridi et al. [39] also stated that the increase in mothers’ perceived susceptibility increased their preventive performance for their children’s accidents [39]. Comparing our study’s results with two others, it is evident that the perceived susceptibility of mothers may not have a significant impact on their children’s dental trauma prevention behavior. Rather, it may be more beneficial to focus on other aspects of the health belief model to encourage mothers to engage in preventive measures for their children’s dental trauma.

Perceived severity includes mothers’ mental belief regarding the severity of harm caused to their children by doing or not doing the behavior. While high perceived severity can induce fear, which can either hinder or motivate preventive behaviors [19], our study found no relationship between perceived severity and mothers’ preventive actions. This may be because mothers view dental trauma as a non-threatening condition. In addition, perceived severity was found to be a less influential factor, possibly because mothers considered dental trauma as a non-health-threatening condition. However, perceived severity remains important because if mothers see dental trauma as a threat, they are more likely to take preventive action. The study by Mahmoodi, reported that due to the educational intervention for mothers’ preventive behaviors to prevent children’s accidents and incidents, perceived severity did not change and was not significant [42]. However, Kamal et al. [37] pointed out that perceived severity had a positive effect on mothers’ preventive health behaviors [37]. Askelson et al. [43], also found that perceived severity had a stronger influence than perceived susceptibility on children’s preventive dental visits [43].

In many cultures, dental trauma is not perceived as a severe or life-threatening condition compared to other childhood injuries (e.g., fractures or arm trauma) [44, 45]. Mothers may prioritize preventing acute medical emergencies over dental injuries, which are often viewed as manageable or cosmetic issues [24, 46]. This cultural minimization of oral health risks could explain the lack of correlation between perceived severity/susceptibility and preventive actions.

HBM constructs may interact differently for dental trauma compared to other health behaviors. For example, perceived barriers (e.g., financial costs, time constraints) and self-efficacy might overshadow severity/susceptibility in preventive behaviors, as our results indicated. Unmeasured variables, such as access to dental care, could mediate these relationships. Mothers with limited resources may focus on immediate health threats rather than preventive measures for dental trauma, regardless of their beliefs.

While HBM is a robust framework, its constructs’ predictive power may vary by context. For dental trauma, interventions should prioritize reducing barriers (e.g., cost, time) and enhancing self-efficacy through hands-on training, as these factors dominated mothers’ behavioral decisions. Future studies could explore cultural narratives around oral health and include moderators like socioeconomic status to refine HBM’s utility in this context.

### Strengths and limitations

This study has several limitations. First, the exclusion of mothers of children with disabilities may introduce selection bias, as their perspectives and challenges regarding dental trauma prevention likely differ from our study population, potentially affecting generalizability. Second, in this study, we only excluded individuals who were unable to read the questionnaires or provide responses due to illiteracy. While this approach ensured data quality, it may somewhat limit the generalizability of findings to low-literacy populations. Future studies could employ alternative methods such as oral interviews, face-to-face surveys, or multimedia tools to facilitate participation from these groups. Third, the reliance on self-reported data makes the findings susceptible to recall bias (inaccurate memory of past behaviors) and social desirability bias may have influenced responses, particularly regarding reported preventive behaviors. While we ensured anonymity to minimize this effect, mothers might still have over-reported ‘ideal’ behaviors. Future studies could benefit from incorporating observational measures to complement self-reports. Fourth, recruitment from a single urban center limits the applicability of results to broader populations, particularly those in rural settings or with diverse healthcare access. These limitations suggest caution in interpreting and generalizing the findings. While the HBM framework and large sample size are strengths.

## Conclusion

The findings of this study demonstrate that maternal knowledge, self-efficacy, and perceived barriers play a decisive role in preventing dental trauma in children. To improve this situation, it is recommended to implement community-based educational programs in healthcare centers and schools, emphasizing the use of mouthguards, seatbelt use, and avoidance of risky behaviors. Additionally, practical workshops for maternal empowerment, subsidized distribution of mouthguards, and integration of preventive education into the healthcare system could prove effective. Further research is also recommended to examine the impact of socioeconomic factors and evaluate the effectiveness of educational interventions. Overall, implementing targeted, multidimensional strategies can significantly contribute to enhancing preventive behaviors and reducing dental trauma among children.

## Data Availability

The datasets used and/or analyzed during the current study are available from the corresponding author on reasonable request.
